# Functional metagenomics reveals abundant polysaccharide-degrading gene clusters and cellobiose utilization pathways within gut microbiota of a wood-feeding higher termite

**DOI:** 10.1038/s41396-018-0255-1

**Published:** 2018-08-16

**Authors:** Ning Liu, Hongjie Li, Marc G. Chevrette, Lei Zhang, Lin Cao, Haokui Zhou, Xuguo Zhou, Zhihua Zhou, Phillip B. Pope, Cameron R. Currie, Yongping Huang, Qian Wang

**Affiliations:** 1 Key Laboratory of Insect Developmental and Evolutionary Biology, Institute of Plant Physiology and Ecology, Shanghai Institutes for Biological Sciences, Chinese Academy of Sciences, Shanghai, China; 2 Key Laboratory of Synthetic Biology, Institute of Plant Physiology and Ecology, Shanghai Institutes for Biological Sciences, Chinese Academy of Sciences, Shanghai, China; 3 Zhejiang Academy of Agricultural Sciences, Hangzhou, China; 4 Department of Energy Great Lakes Bioenergy Research Center, Wisconsin Energy Institute, University of Wisconsin-Madison, Madison, WI USA; 5 Department Bacteriology, University of Wisconsin-Madison, Madison, WI USA; 6 Department of Genetics, University of Wisconsin-Madison, Madison, WI USA; 7 Institute for Synthetic Biology, Shenzhen Institutes of Advanced Technology, Chinese Academy of Sciences, Shenzhen, China; 8 Department of Entomology, University of Kentucky, Lexington, USA; 9 Faculty of Chemistry, Biotechnology and Food Science, Norwegian University of Life Sciences, Ås, Norway; 10 Shanghai First Maternity and Infant Hospital, Tongji University School of Medicine, Shanghai, China

**Keywords:** Microbial ecology, Applied microbiology, Microbial ecology, Applied microbiology

## Abstract

Plant cell-wall polysaccharides constitute the most abundant but recalcitrant organic carbon source in nature. Microbes residing in the digestive tract of herbivorous bilaterians are particularly efficient at depolymerizing polysaccharides into fermentable sugars and play a significant support role towards their host’s lifestyle. Here, we combine large-scale functional screening of fosmid libraries, shotgun sequencing, and biochemical assays to interrogate the gut microbiota of the wood-feeding “higher” termite *Globitermes brachycerastes*. A number of putative polysaccharide utilization gene clusters were identified with multiple fibrolytic genes. Our large-scale functional screening of 50,000 fosmid clones resulted in 464 clones demonstrating plant polysaccharide-degrading activities, including 267 endoglucanase-, 24 exoglucanase-, 72 β-glucosidase-, and 101 endoxylanase-positive clones. We sequenced 173 functionally active clones and identified ~219 genes encoding putative carbohydrate-active enzymes (CAZymes) targeting cellulose, hemicellulose and pectin. Further analyses revealed that 68 of 154 contigs encode one or more CAZyme, which includes 35 examples of putative saccharolytic operons, suggesting that clustering of CAZymes is common in termite gut microbial inhabitants. Biochemical characterization of a representative xylanase cluster demonstrated that constituent enzymes exhibited complementary physicochemical properties and saccharolytic capabilities. Furthermore, diverse cellobiose-metabolizing enzymes include β-glucosidases, cellobiose phosphorylases, and phopho-6-β-glucosidases were identified and functionally verified, indicating that the termite gut micro-ecosystem utilizes diverse metabolic pathways to interconnect hydrolysis and central metabolism. Collectively, these results provide an in-depth view of the adaptation and digestive strategies employed by gut microbiota within this tiny-yet-efficient host-associated ecosystem.

## Introduction

Termites are remarkably efficient at degrading recalcitrant woody biomass. They digest 74‒99% of dietary cellulose hourly via their symbiotic gut microbiota [1–3], thus having a significant impact on carbon cycling in (sub)tropic forests. With resurgent biotechnological interests, the past decade of termite gut “omics” studies have identified abundant carbohydrate-active enzymes (CAZymes) involved in plant biomass deconstruction [4–9], which has significantly advanced our understandings of symbiotic digestion in these gut ecosystems.

For the evolutionary basal wood-feeding “lower” termites, wood particle digestion is accomplished by a dual cellulolytic system that combines enzymes of both host and its intestinal symbionts including cellulolytic flagellates and bacteria [10–15]. For “higher” wood-feeding termites (family Termitidae), which are entirely free of gut flagellates, plant biomass is digested within their intestinal tract via associations with “prokaryotic” microbiota, which is hypothesized and increasingly corroborated for a role in symbiotic digestion for the termite hosts. Metagenomic studies on *Nasutitermes* species revealed that Spirochetes of the genus *Treponema* and Fibrobacteres lineages contribute the vast majority of genes encoding putative CAZymes in the hindgut [4, 6], and detailed studies on these termites revealed fiber-associated bacteria from these lineages possessing substantial cell-bound cellulolytic activity [16–18]. Studies have also shown that the prevalent *Treponema* lineages in higher wood-feeding termite gut are highly diverse and mostly as-yet uncultured [19]. It has also been estimated that as many as a hundred *Treponema* species could be present in the gut of a single termite species [20]. Collectively, the high diversity and metabolic versatility of these as-yet uncharacterized lineages makes speculations concerning their functional role problematic.

Despite increased efforts to understand digestion in higher wood-feeding termites, the mechanisms underlying the highly efficient polysaccharide utilization by gut microorganism largely remain unclear. In particular, little is known about their fibrolytic gene organizations and enzymology [21]. Two main paradigms regarding fibrolytic gene clusters encoding multi-enzyme complexes have been widely explored, namely the cellulosome system and the polysaccharide utilization loci-like systems (PULs) [22]. These are considered efficient plant biomass degradation systems widespread in bovine rumen, Tammar wallaby, human distal gut and ocean environment [23–27]. Whereas in termite guts, both cellulosomes and PULs remain either poorly represented or elusive to capture by previous shotgun sequencing studies. Thus, it is apparent that there exists yet to be discovered degradative strategies that are employed for rapid lignocellulose deconstruction in these tiny intestinal ecosystems [1].

Using a combination of large-scale functional screens, pyrosequencing, and enzymology, we provide new insights into the biochemical activity and modular architecture relevant to the diverse glycan-degrading enzymes in the gut microbiota of an unexplored wood-feeding termite *G*. *brachycerastes* (family: Termitidae subfamily: Termitinae). Specifically, we: (i) cloned metagenomic DNA from termite gut into fosmid vectors and conducted functional screens of 50,000 fosmid clones; (ii) sequenced 173 of the retrieved total 464 positive clones with lignocellulytic activities; (iii) identified putative CAZyme genes through in silico analyses; (iv) discovered abundant polysaccharide-degrading gene clusters and cellobiose utilization pathways; (v) functionally analyzed a xylanase cluster and (vi) functionally verified diverse cellobiose-degrading enzymes.

## Materials and methods

### Chemicals and reagents

Kod Plus DNA Polymerase, restriction endonucleases, and T4 DNA ligase were purchased from Takara (Japan). The AxyPrep^TM^ DNA gel extraction kit, AxyPrep^TM^ plasmid miniprep kit, and AxyPrep^TM^ PCR cleanup kit were obtained from Axygen (USA). A fosmid library was constructed with the vector of pCC2FOS™ (Epicentre, USA) and the host strain of EPI300™-T1^R^ (Epicentre, USA). Screening substrates carboxymethyl cellulose, 4-methylumbelliferyl-b-D-cellobioside (4-MUC), esculin hydrate, ferric ammonium citrate, and birchwood xylan were all purchased from Sigma-Aldrich (USA). Selected functional genes were cloned in *Escherichia coli* Top10 (Novagen, USA) and expressed in *E. coli* BL21 (Novagen, USA) with the vector of either pET-28a(+) or pET-22b(+) (Novagen, USA). Xylooligosaccharides used as standards for TLC, including xylose (Sigma, USA), xylobiose (Wako, USA), xylotriose (Wako, USA), xylotetraose (Megazyme, Ireland), xylopentose (Megazyme, Ireland), and xylohexaose (Megazyme, Ireland) were purchased from Express Technology Co., Ltd. D(+)-Cellobiose (Wako, USA) used as substrate for phosphorylase activity assay was purchased from Express Technology Co., Ltd. Cellobiose-6-phosphate used as substrate for 6-phospho-beta-glucosidase activity assayed was provided by Prof. Congzhao Zhou from University of Science and Technology of China and Prof. Jack Thompson from National Institute of Dental and Craniofacial Research, NIH.

### Termite sampling and intestinal microbial DNA extraction

One *Globitermes* colony harboring worker and soldier termites was collected in March 2008 from a forest area in Xishuangbanna, Yunnan Province, China. The soldier termites were subjected to both morphological and mitochondrial COII gene-based molecular identification [28], confirming this colony was *G. brachycerastes*. Surface sterilization and dissection of the whole gut was performed as described previously [29]. Whole gut samples were collected and immediately frozen in liquid nitrogen and stored at −80 °C until use. Metagenomic DNA from about 100 whole guts of adult workers was extracted using a modified indirect method and quantified, as described previously [8, 9], to yield sufficient amount DNA for direct construction of a fosmid library. In brief, a mild trypsin digestion step was firstly applied to disintegrate the termite gut tissues and release the microbial cells. Then an 800 × *g* centrifugation step was performed to remove gut tissues and cells. Finally, the collected microbial cells were subjected to DNA extraction.

### Bacterial community composition analysis

The V3 region of bacterial 16S rRNA genes were amplified with the forward primer P2 (5′-ATTACCGCGGCTGCTGG-3′) and the reverse primer P3 (5′-GC CGC CCG CCG CGC GCG GCG GGC GGG GCG GGG GCA CGG GGG GCC TAC GGG AGG CAG CAG-3′) [30] from 10 ng of extracted DNA. PCR was performed, as previously described [28]. Amplicons were pyrosequenced (454 GS FLX with Titanium technology, Roche) at the Chinese National Human Genome Center in Shanghai, China. The pyrotag sequences were denoised using Acacia (version 1.52), chimeras removed using UCHIME and quality filtered using stringent conditions (reads >200 bp, no ambiguous bases, and a maximum number of homopolymers ≤8, Q cutoff at quality score 25) [31]. The quality-checked sequences were then aligned using the Mothur software suite (version 1.29.0 [32]). To improve taxonomic resolution, the sequences were classified against the manually curated reference database DictDb 3.0 [29, 33], which consists of the SILVA non-redundant database supplemented with numerous unpublished sequences from termite and cockroach guts.

### Fosmid library preparation and identification of fosmid clones bearing cellulases and xylanase genes

Gel purification, electroelution, and concentration of the extracted whole gut metagenomic DNA were performed according to Brady’s protocols [34]. The fosmid library was constructed with the CopyControl™ pCC2FOS™ fosmid library production kit (Epicentre, USA), following manufacturer’s instructions. The library contains about 50,000 clones, which were preserved in 130 blocks of 384-well microtiter plate at −80 °C. Functional screen for the four major hydrolases activities (endoglucanase, exoglucanase, β-glucosidase and endoxylanase) was performed, as described previously [8]. In brief, by using a 384-well inoculating needle holder, each 384-well plate was manually printed onto four plates with 12.5 µg/ml of chloramphenicol and different screening substrates, respectively (Figure S1).

### Fosmid sequencing and analysis of the functional genes

Based on functional screening, a total of 173 positive fosmid clones were randomly selected from variant levels of halo against screening substrates for full sequencing (Figure S1), including 68 endoglucanase-, 15 exoglucanase-, 40 β-glucosidase-, and 50 endoxylanase-positive clones (10 out of these clones exhibited dual enzyme specificities). After cell density normalization, every 10 to 12 different fosmids were pooled as a sample for the whole fosmid DNA extraction using the QIAGEN Large-Construction kit according to manufacturer’s instructions. Extracted DNA was directly subjected to 454 pyrosequencing (Roche Diagnostics, USA). After assembly (Newbler 2.5.3), coding regions and bacterial operons were predicted by FGENESB (http://linux1.softberry.com/berry.phtml). The predicted amino acid sequences were aligned to the eggNOG v4.5.1 database [35] and KEGG for further functional prediction (E-value <1.0e^−5^). Searches for carbohydrate-active enzymes was performed against dbCAN database with default parameters. Signal peptides were predicted by SignalP 4.1 in CBS (http://www.cbs.dtu.dk/services/SignalP/). Molecular masses and isoelectric points were predicted by ExPASy (http://www.expasy.ch/tools/protparam.html). Protein cellular location was predicted using CELLO v.2.5 (http://cello.life.nctu.edu.tw) [36].

### Cloning and heterologous expression of one xylanase cluster and three types of cellobiose-metabolizing genes

To better understand biochemical properties of lignocellulose-degrading gene clusters and cellobiose-metabolizing genes derived from this fosmid library, several genes have been verified before including two β-glucosidases (GH1, on contig00059, GenBank accession no.: JQ844187 and GH1, on contig00057, GenBank accession no.: JN903693.1) [37, 38], two xylanses (GH10, on contig00057, GenBank accession no.: JN903693.1 and GH11, on contig00059, GenBank accession no.: KJ450881) [39, 40], and 8 β-xylosidases (GH1, GH3 and GH43, GenBank accession no.:KY618667-KY618674) [41]. In the present study, a total of 14 genes distributed in 6 contigs were subjected to prokaryotic cloning, and phylogenetic and functional analysis, which including one typical xylanase (GH10) cluster on contig00057 together with members of two types of cellobiose-metabolizing genes distributed in GH1, GH4 and GH94 (see details in Table S1). All genes, removing the signal peptide coding sequence (predicted by SignalP 4.1), were amplified from the fosmid DNA by 30 cycles of PCR using the primers listed in Table S1, accordingly. PCR products were purified with the AxyPrep^TM^ PCR cleanup kit, digested with double restriction enzymes, and ligated into pET-28a(+) or pET-22a(+) treated with the same sets of restriction enzymes. The recombinant plasmids were transformed into *E. coli* BL21, which was grown on LB plate (50 µg/ml kanamycin for the pET-28a(+) vector; 50 μg/ml ampicillin for the pET-22a(+) vector) at 37 °C (all the detailed plasmids and strains present in Table S1). Induction, purification and quantification of the recombinant proteins were carried out by following the same procedures as described in our earlier report [8], except all proteins were washed with a gradient of 20 mM, 35 mM and 50 mM imidazole to exclude background proteins before eluted with 250 mM imidazole.

### Enzymatic assays

Enzymatic assays were performed to characterize the biochemical properties of xylanase cluster and to test the activity of cellobiose-metabolizing enzymes. For the xylanase cluster, effects of pH and temperature on xylanase activity and stability, saturate substrate concentration, Km, and Thin-layer chromatography (TLC) for hydrolysis products were determined, as described previously [8]. For cellobiose-metabolizing enzymes, since one representative of β-glucosidase from GH1 on contig00059 has been cloned and functionally characterized in our previous work [37], only cellobiose phosphorylase and 6-phospho-beta-glucosidase genes were subject to functional verification by using a reducing-sugar assay. Cellobiose phosphorylase activity was determined by detecting the formation of glucose from cellobiose according to Sasaki et al. [42] with slight modifications. The reaction mixture, in a total volume of 0.1 ml, consisted of 50 mM Tris/HCl buffer, pH 7.5, 5 mM MgCl_2_, 5 mM H_3_PO_4_, 5 mM cellobiose, and 50 μl of crude enzyme solution, with glucose instead of enzyme as positive control and no enzyme as negative control. After incubation at 37 °C for 2 h, cellobiose hydrolysis was measured by the release of glucose as detected using a Glucose (GO) Assay Kit (Sigma-Aldrich) [43]. 6-phospho-beta-glucosidase activity was assayed by detecting the formation of glucose from 6-P-cellobiose according to Palmer’s method with modifications [44]. The reaction mixture, in a total volume of 0.1 ml, consisted of 50 mM Hepes buffer, pH 7.5, 1 mM MgCl_2_, 1 μM NADP^+^, 0.5 mM 6-P-cellobiose, and 50 μl of crude enzyme solution, with glucose instead of enzyme as positive control and no enzyme as negative control. After incubation at 50 °C for 2 h, 6-P-cellobiose hydrolysis was also monitored by the release of glucose as detected using a Glucose (GO) Assay Kit (Sigma-Aldrich) [43].

### Nucleotide accession number

The raw pyrosequencing data of the V3 regions of 16S rRNA genes amplified from termite gut of *G*. *brachycerastes* were deposited at NCBI under accession SAMN08040946. The GenBank accession number for the fosmid contig with CAZyme is JN903693, JQ844164-JQ844243, JQ844245-JQ844278, JQ844280-JQ844301, MG852068-MG852084. The raw data for all the fosmid contig under SRA access no.: SRP125254.

## Results and discussion

### Bacterial diversity

Pyrosequencing of the V3 region of the bacterial 16S rRNA genes amplified from the termite whole gut yielded 1256 high quality reads, which were further taxonomically classified against the manually curated reference database DictDb 3.0. Of the 10 phyla represented in the dataset, the majority of sequences were Spirochaetes (77.23%), followed by Firmicutes (4.3%), Fibrobacteres (3.98%), Bacteroidetes (2.63%), and Candidate_phylum_TG3 (2.31%) (Table 1, see details in Table S2, which contains an interactive spreadsheet with the detailed classification results for all the taxonomic ranks (down to genus)). In general, this bacterial community structure largely resembles those of higher wood-feeding *Nasutitermes* termites at the phylum level, with higher abundance of Spirochaetes and Fibrobacteres [4, 6, 45, 46], while fundamentally different to what is commonly found in fungus, humus and soil feeders with a higher prevalence of Firmicutes [29, 46, 50]. Nonetheless, unlike the dominant *Treponema* Cluster Ic and If in *Nasutitermes* spp. termite guts [45–47], the most prevalent spirochetes in this *G*. *brachycerastes* were the unclassified *Treponema* Cluster I (Table S2). Our results suggest that the intestinal bacterial community is structured not only to the hosts dietary specialization, but it also differs considerably with regards to the genus-level bacterial lineages that are found within the same dietary habits. This is consistent with the recent proposed mixed-mode transmission as the driving force shaping the gut community of termites [48, 49].

**Table 1:** Community composition of *G*. *brachycerastes* revealed by V3 region of 16S rRNA genes

Phylum	Reads of V3	Percentage
Spirochaetes	970	77.23
Firmicutes	54	4.30
Fibrobacteres	50	3.98
Bacteroidetes	33	2.63
Candidate_phylum_TG3	29	2.31
Proteobacteria	13	1.04
Chlorobi	10	0.80
Synergistetes	5	0.40
Acidobacteria	4	0.32
Candidate_phylum_SR1	1	0.08
unclassified	87	6.93
	1256	100

### Function-based screening and pyrosequencing revealing the adaption of woody diet in the *Globitermes* termite gut metagenome

Activity-based screening was performed against four classes of lignocellulolytic activity, from approximately 50,000 fosmid clones that were constructed from termite gut metagenomic DNA. This effort resulted in a total of 464 positive clones, including 267 endoglucanase-positive clones, 24 exoglucanase-positive clones, 72 β-glucosidase-positive clones and 101 endoxylanase-positive clones. A total of 173 positive fosmid clones with dual enzyme specificities or variant levels of activity, were selected for 454 pyrosequencing (Figure S1). After assembly and annotation, 611 contigs comprising 4.67 Mbp data were obtained, which included 154 contigs-encoding predicted CAZymes. A total of 219 putative CAZyme genes from 30 different CAZy families were identified within the *Globiterms* termite gut microbiome dataset (Table 2 and Table S3). Particularly, our findings suggest an enrichment of glycoside hydrolases that attack cellulose and backbones of hemicellulose in *G. brachycerastes*, including members from GH3, GH5, GH10 and GH11, which largely resembles that of the wood-feeding higher termites *Nasutitermes* spp. [4, 6]. In contrast, the *G. brachycerastes*-CAZyme profile greatly differs from those observed in the microbiome of fungus-cultivating termites *Odontotermes yunnanensis* and *Macrotermes natalensis* [9, 50] and dung-feeding higher termite *Amitermes wheeleri* [4], in which enriched families tended to be involved in the breakdown of cello-oligosaccharides and short and side chains of hemicellulose. These results indicate that the functional profiles of the termite microbiome are likely associated with feeding guilds (Table S4). Although many termite microbiomes have already been explored for lignocellulolytic genes, all the putative CAZymes from the unexplored Termitinae subfamily were evolutionarily distant to representatives in the current NCBI nr databases (identity from 29.5 to 89%) (see Figure S2 for phylogenetic analysis of main GH families and Table S5 for identity). The diverse sets of mostly novel CAZymes and unique microbial lineages archived here support the hypothesis that a strong phylogenetic relationship between bacterial microbiota and host among the higher termite lineages [51].

**Table 2:** Statistics of putative plant fibrolyitc genes from 173 sequenced fosmids

CAZy family	Known activity	Gene No.
Cellulases family		
GH1	beta-glucosidase, 6-phopho-beta-glucosidase	11
GH3	beta-glucosidase, beta-D-xylosidase, beta-N-acetylhexosaminidase	31
GH4	6-phopho-beta-glucosidase	1
GH5	endoglucanase	65
GH9	endoglucanase	7
GH44	endoglucanase	1
GH45	endoglucanase	2
GH94	cellobiose phosphorylase, chitobiose phosphorylase	12
Hemicellulases family		
GH2	beta-mannosidase	2
GH8	endo-1,4-beta-xylanase	4
GH10	endo-1,4-beta-xylanase	24
GH11	endo-1,4-beta-xylanase	18
GH26	beta-mannanase	3
GH29	α-L-fucosidase	1
GH30	glucuronoarabinoxylan endo-1,4-beta-xylanase	3
GH31	α-xylosidase	2
GH39	xylan 1,4-beta-xylosidase	2
GH43	alpha-N-arabinofuranosidase	6
GH51	α-L-arabinofuranosidase	1
GH53	arabinogalactan Endo-1,4-beta-galactosidase	2
GH67	alpha-glucuronidase	1
GH74	xyloglucanase	4
GH95	alpha-L-fucosidase	3
GH115	xylan α-1,2-glucuronidase	1
CE1	acetyl xylan esterase	4
CE4	acetyl xylan esterase	1
CE6	acetyl xylan esterase	1
Pectinase family		
GH105	unsaturated rhamnogalacturonyl hydrolase	2
PL1	pectate lyase	3
PL11	rhamnogalacturonan endolyase	1

Despite the detection and sequencing of selected exoglucanase-positive clones through functional screening, no putative cellobiohydrolases genes were found from the subsequently generated contigs. In fact, exo-cellulases are relatively rare among bacterial cellulolytic systems, with only a few reported cases mainly attributed to the cellulosomal bacteria affiliated to the Clostridiales [52]. Exo-cellulases are particularly scarce in anaerobic gut metagenomic datasets, with limited number of GH48s cellobiohydrolases observed in several rumen microbiomes [53, 54]. While in termite guts, exo-cellulases have only been found from protists of lower termites and appear to be absent in protist-free higher termite microbiomes [1, 14]. Although characterized representatives of exo-cellulases are seldom observed, they are still believed to be necessary for microbes to effectively extract energy and carbon sources from ingested lignocellulosic materials. Possible explanations for the failed annotation of known cellobiohydrolase genes to accompany the observed exo-cellulase fosmid activity observed in this study, could be that they are located on the missing fractions of the sequenced fosmids or they are encoded by novel ORFs that do not match known CAZy families.

### Discovery of abundant lignocellulose-degrading gene clusters in the sequenced fosmid clones

**Fig. 1:**
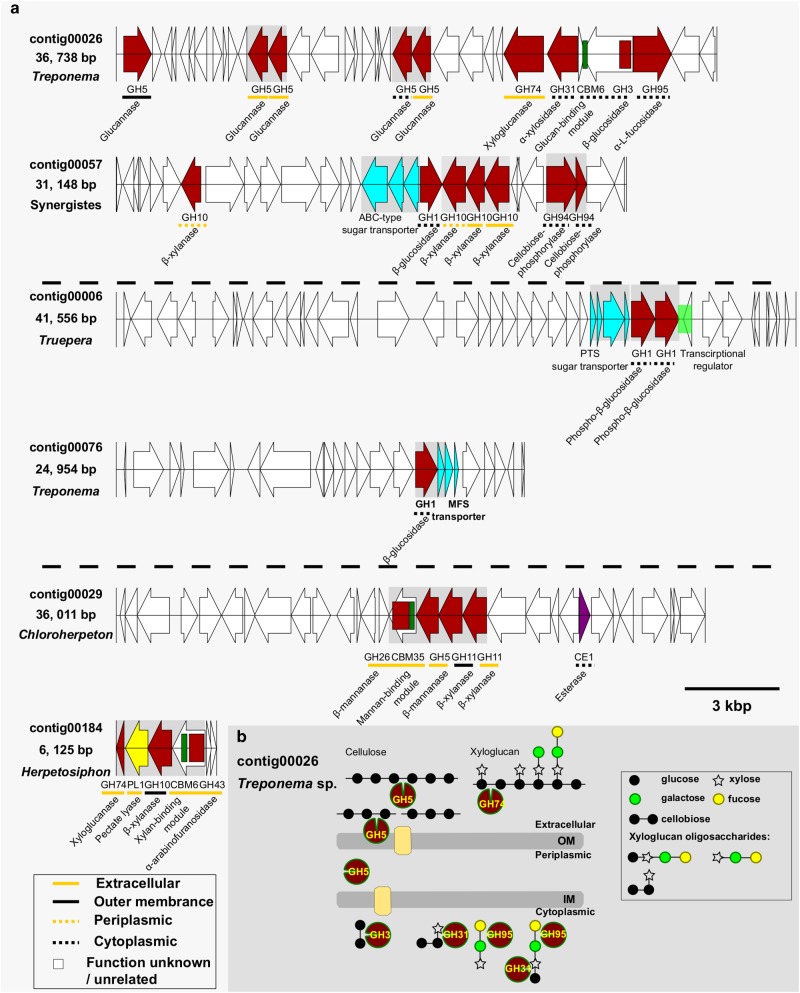
Putatively representative fibrolytic gene clusters and a hypothetical model recovered from gut microbiome of *G. brachycerastes*. **a** Gene organization of the representative fibrolytic gene clusters targeting plant polysaccharides. Shading box indicates shared operon. **b** A hypothetical model of contig00026 depicts complete cellulose and hemicellulose cleavage pathways (see the text for more details). Proteins marked with an asterisk. OM outer membrane, IM inner membrane

Using a large set of fosmid contigs, much longer fragments of genomic information were retrieved, enabling examination of the fibrolytic gene organization. Bioinformatic analysis of the 154 contigs (average length 20.557 Kb, Table S6) with genes encoding putative CAZymes revealed that 68 of these contigs possess more than one hydrolase gene (Table S7). Many CAZymes from the same or different CAZy families, were arranged in putative operons with related carbohydrate binding domains and/or substrate transporters (Fig. 1). Furthermore, the predicted substrate-specificity of the different CAZy families encoded in these operons suggest varying putative saccharolytic activities against cellulose, mannan, xylan, and pectin. Specific contigs featured extensive arrays of GHs targeting the various backbone and side chain linkages found in various hemicellulose polysaccharides, including arabinoxylan, xyloglucan, xylan, and mannan. In particular, contig00026 was found to tandemly encode genes involved in xyloglucan degradation, including a GH74 xyloglucanase, a GH31 α-xylosidase and a GH95 α-L-fucosidase (Fig. 1a). Contig00029 encoded a xylan- and mannan-decomposing operon including a GH26 β-mannanase that is appended to a putative mannan-binding CBM35, a GH5 β-mannanase, a CE1 esterase, and two GH11 β-xylanases. Contig00184 encoded an operon including a GH10 β-xylanase and GH74 xyloglucanase that are both suspected to cleave backbone linkages. Debranching CAZymes were also noted such as a GH43 α-arabinofuranosidase that is appended to a putative xylan-binding CBM6, as well as a PL1 pectate lyase.

Despite early “omic” reports on numerous fibrolytic genes within the wood-feeding lower and higher termites [6, 55–57], the gene arrangements that confer this fibrolytic lifestyle basically remain unknown. The findings presented in this study indicate that within a wood-diet adapted termite gut microbiome, functionally relevant genes targeting specific plant cell-wall polysaccharides tend to aggregate or form putative operons. This pattern is reminiscent of the reported polysaccharide utilization loci-like systems (PULs) in mammalian gut systems and environmental samples [23–25]. In human gut bacterial symbionts, it has been shown that complex oligosaccharides provide regulatory cues that activate PUL operons and that each PUL operon is highly specific for a defined plant cell-wall polymer [25], implying that bacterial symbionts adapt to different carbohydrate niches by evolving genes that target unique suites of available polysaccharides. Presumably, similar mechanism may be employed in the termite gut microbiome, where the intake of complex cell wall polysaccharides are possible regulatory cues that trigger expression of the inherent gene clusters.

To assess which microbial groups contribute functional enzymes for lignocellulose degradation in the given wood-feeding termite gut ecosystem, taxonomic origins of the 154 contigs that carry CAZyme genes were predicted by PhyloPythiaS [58]. Spirochaetes (*Treponema*) and Firmicutes contributed 20.1% each of the contigs bearing CAZy genes, followed by members of Synergistetes (19.5%) and Proteobacteria (16.2%) (Table S7).

### Recovery of novel CAZyme diversity and distribution in a single *Treponema* sp.

The speculations concerning the functional roles of mostly uncultured *Treponema* in termites are problematic, as members of this lineage are both highly diverse and metabolically versatile. The few existing isolates from lower termites either can ferment glucose and cellodextrins (*T. azotonutricium*), carry out reductive acetogenesis from H_2_ and CO_2_ (*T. primitia*) [59–62], perform nitrogen fixation [63] or even hold the potential for aromatic-ring cleavage (*T. primitia*) [64]. In the P3 lumen of the higher *Nasutitermes* sp. termite, the abundance of glycoside hydrolases is correlated with Spirochaetes in metagenomic datasets [4, 6]. Further, they are found associated with wood particles [18], suggesting spirochetes may also contribute to fiber digestion in termite guts. In the present study, we localized *Treponema*–affiliated gene clusters representing complete cleavage pathways that putatively target cellulose–xyloglucan complexes that are found in the plant cell-wall (GH5s, GH3, and CBM6 for cellulose, and GH74, GH31, and GH95 for hemicellulose) (Fig. 1b). Xyloglucans are a major hemicellulose component in the plant primary cell wall (10% of woody cell-wall) [65] and are able to cross-link with cellulose microfibrils and lead to the formation of cellulose-xyloglucan complex in woody biomass. The xyloglucan/cellulose-degrading cluster observed in this study together with its ability to assist depolymerization of wood fragments in the termite hindgut [18], suggests that *Treponema*-affiliated species are capable of removing the physical structural protection of xyloglucan prior to gaining access and degrading beta-1,4-glucan linkages, which likely underscores the wood-degrading process in termite gut.

To further investigate sequence similarity and gene cluster structure conservation, we compared contig00026 to the publically available *Tremponema* genomes (*n* = 181), including three strains from termite guts: *T. primitia* ZAS-1, *T. primita* ZAS-2, and *T. azotonutricium* ZAS-9 [60–62, 66]. Specifically, no conserved synteny was found between contig00026 and these genomes, and the majority of blastp hits were <45% identity, indicating that the operon encoded in contig00026 constitutes a genomic fragment affiliated to an as-yet undescribed *Tremponema* lineage.

### Biochemical characterization of a xylanase cluster on Contig00057 reveals distinct physicochemical properties and hydrolysis patterns

**Fig. 2:**
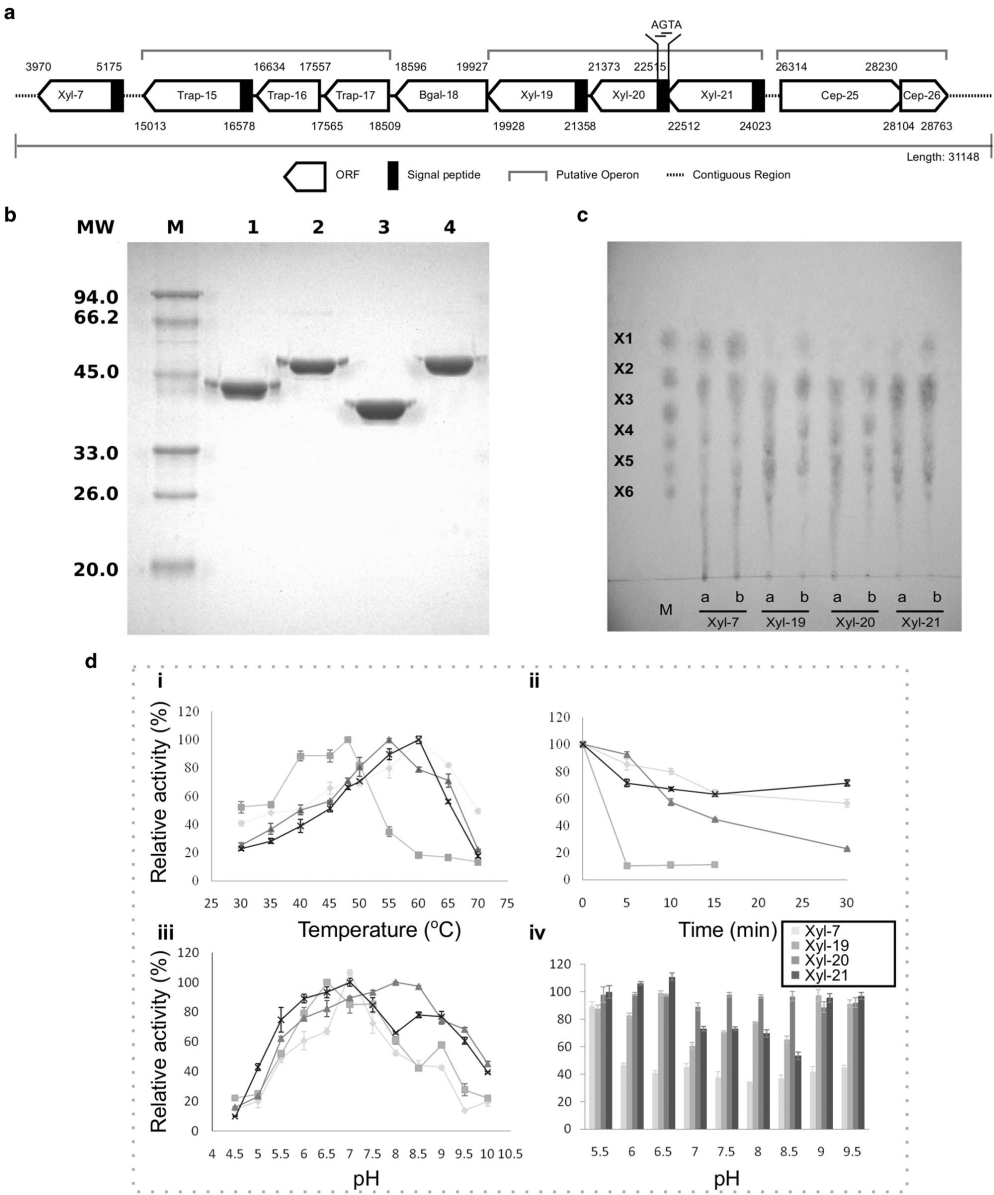
Biochemical characterization of the four xylanases on the fosmid contig00057. **a** Schematic organization of lignocellulase genes on contig00057. **b** 12% SDS–PAGE analysis of the four purified xylanases. Lanes: M, protein molecular weight marker; lane 1–4: purified recombinant proteins for Xyl-ORF7, Xyl-ORF19, Xyl-ORF20 and Xyl-ORF21, with molecular weights of 45.1, 51.3, 42.3 and 53.9 kDa, respectively. **c** TLC analysis. Marker (M), xylose (X1), xylobiose (X2), xylotriose (X3), xylotetraose (X4), xylopentose (X5), and xylohextose (X6). **a** hydrolytic products of 10 min of the four xylanases against birch wood xylan; **b** hydrolytic products of 12 h of the four xylanases against birch wood xylan. **d** pH and temperature profile. (i) Temperature range of Xyl-ORF7, Xyl-ORF19, Xyl-ORF20 and Xyl-ORF21 were assayed between 30 and 70 °C. (ii) Thermostability were measured by dectecting the residual activity after preincubation at 50 °C for 5 min, 10 min, 15 min, and 30 min respectively. (iii) pH range were assayed between pH 4.5 and 10. (iv) pH stability were examined after preincubation in buffers ranging from pH 4.5 to 10 at 4 °C for 5 days. All activity assays were obtained from triplicate experiments

Among these large fosmid inserts, a 31 Kb fragment (JN903693) exhibited a high density of genes encoding fibrolytic related enzymes, including one beta-glucosidase, two cellobiose phosphorylases, three sugar ABC transporter proteins and four xylanases (Fig. 2a). Homology searches indicated that Contig00057 originated from none of the existing sequenced bacterial genomes available in NCBI and were predicted by PhyloPythiaS to be of Synergistes origin. Sequence analysis of the four xylanases revealed that they all belong to GH10, but they have relatively small molecular mass (45.1, 51.3, 42.3, and 53.9 kD, respectively) compared to typical members of this family, which may be due to the general lack of carbohydrate binding modules (CBMs). All four also encode predicted signal peptides at the N-terminus (Table S1) and were predicted by CELLO as either extracellular or periplasmic proteins. Homology searches further showed that they had only 38 to 43% protein sequence identity with existing members in the database and between 34.9 and 56.1% protein sequence identity among themselves.

Biochemical characterizations of all the four clustered GH10 xylanases (Xyl-ORF7, Xyl-ORF19, Xyl-ORF20 and Xyl-ORF21) were conducted to provide insights into their roles in xylan degradation, which covering the wood-feeding higher termites’ biological temperature range 27–30 °C and pH 6–10 [45]. All four recombinant proteins were purified on a Ni-NTA column (Qiagen) with the presence of an N-terminal 6^*^His-tag and then followed SDS–PAGE analysis demonstrated the molecular weights were in agreement with predictions (Fig. 2b). Thin-layer chromatography (TLC) analysis of hydrolytic products of the four xylanases against birchwood xylan showed that the three clustered xylanases (Xyl-ORF19, Xyl-ORF20 and Xyl-ORF21) released mainly xylooligosaccharides with almost no trace of xylose except over extended periods of hydrolysis, while Xyl-ORF7 was able to release xylose monomer as one of the main hydrolytic products besides xylooligosaccharides (Fig. 2c). The analysis of temperature effect on the four xylanases revealed that Xyl-ORF7, Xyl-ORF19, Xyl-ORF20 and Xyl-ORF21 had optimal activity at 60, 48, 55, and 60 °C and retained >80% of activity at 55–65, 38–50, 50–60, 52–62 °C, respectively (Fig. 2d i). Thermostability was assessed after incubating at 50 °C or above for 5 min and Xyl-ORF7, Xyl-ORF19, Xyl-ORF20 and Xyl-ORF21 could retain about 50%, 0%, 20%, and 70% of their maximum activity, respectively (Fig. 2d ii). The pH profiles of the four xylanases also showed considerable divergence. Xyl-ORF7, Xyl-ORF19, Xyl-ORF20 and Xyl-ORF21 had pH optimum of 7, 6.5, 8 and 7, respectively and exhibited >60% of highest activity within a pH range of 6–7.8, 5.7–8, 5.5–9.5, and 5.5–9.5, respectively (Fig. 2d iii). The pH stability assay after incubating at pH 5.5 to 9.5 for 5 days at 4 °C revealed that Xyl-ORF7 was stable only at pH 5.5, Xyl-ORF19 and Xyl-ORF21 were more stable at bilateral regions than at the neutral regions, while Xyl-ORF20 could maintain stability over the broad pH range 5.5–9.5 (Fig. 2d iv). Activity assays performed under each of their optimal conditions found that Xyl-ORF7, Xyl-ORF19, Xyl-ORF20 and Xyl-ORF21 had an activity of 264.7 ± 7.4, 113.8 ± 3.8, 112.8 ± 1.0, and 548.7 ± 28.4 U/mg against birchwood xylan, respectively (Table 3). Kinetic analysis showed that their Km values for birchwood xylan were 24.4, 16.2, 7.4, and 1.7 mg/ml, respectively (Table 3), indicating an increasing affinity of each for the substrate. This is in accordance with saturation by a decreasing concentration of 1.8%, 1%, 0.4%, and 0.3% birchwood xylan, respectively (Table 3). Overall, the four GH10 xylanases differ from each other regarding enzyme activity, pH and temperature profiles, substrate affinity, and hydrolysis patterns.

**Table 3:** Biochemical characterizations of four clustered GH10 xylanases (Xyl-ORF7, Xyl-ORF19, Xyl-ORF20, and Xyl-ORF21)

Xylanase	Optimal pH	Optimal temperature (°C)	Saturate substrate concentration (%)	Km (mg/ml)	Activity (U/mg protein)
Xyl-ORF7	7.0	60	1.8	24.4	264.7 ± 7.4
Xyl-ORF19	6.5	48	1.0	16.2	113.8 ± 3.8
Xyl-ORF20	8.0	55	0.4	7.4	112.8 ± 1.0
Xyl-ORF21	7.0	60	0.3	1.7	548.7 ± 28.4

Xylan is one of the most abundant components in wood (10–30% of dry wood), where it intimately interacts with cellulose microfibrils in regions where the microfibrils are amorphous (i.e., non-crystalline). In particular, it plays a role in protecting cellulose and prevents the gradual disruption of the fibrillar network during the hydrolysis process [65]. Herein, the distinct Synergistes-affiliated xylanases observed in this study, are hypothesized to contribute to removal of xylans in the termite gut to assist cellulases gaining access to the microfiber.

### Recovery and functional verification of diverse cellobiose metabolic enzymes

**Fig. 3:**
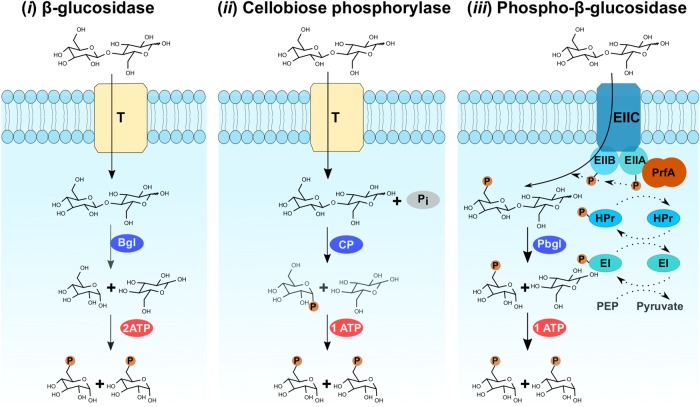
Identification and functional verifications of genes related to three well-known cellobiose-metabolizing pathways in gut microbiome of *G*. *brachycerastes*. T:transporter, Bgl:β-glucosidase, CP: cellobiose phosphorylase, Pbgl:6-phospho-β-glucosidase, Pi:inorganic phosphate, EII:sugar-specific membrane components of PEP-PTS, HPr and EI soluble components of PEP-PTS

Enzymatic degradation of cellulose involves the synergistic action of the upstream endoglucanases and cellobiohydrolases, as well as the downstream saccharifying enzymes. During this cellulose hydrolysis process, cellobiose is one of the most common intermediates, whose accumulation can inhibit upstream cellulases. Therefore, promptly removing cellobiose could effectively improve the entire cellulolytic process [67, 68]. Cellobiose metabolism involves mainly three pathways (Fig. 3). β-glucosidase (EC 3.2.1.21) mediated hydrolytic processes (pathway i), will cleave the beta-1,4 linkage creating two glucose units. Cellobiose phosphorylases (EC 2.4.1.20) will mediate inorganic phosphate-dependent phosphorilytic processes (pathway ii) that cleave intracellular cellobiose into glucose and glucose-1-phosphate (G1P) by using inorganic phosphate (G1P can then be converted to glucose-6-phosphate without the need for ATP by the enzyme phosphoglucomutase) [69]. Finally, 6-P-β-glucosidases (EC 3.2.1.86) will mediate ATP-dependent hydrolytic processes [70, 71] (pathway iii) that enable simultaneous translocating and phosphorylating of cellobiose. From the present *Globiterms* termite gut microbiome, we observed typical cellobiose hydrolase β-glucosidase genes (11 GH1 and 32 GH3), as well as two other atypical cellobiose-degrading gene families, including GH94 cellobiose phosphorylases (*n* = 12) and putative 6-P-β-glucosidases (2 GH1 and one GH4) (Table 2 and Table S2). Indeed, cellobiose phosphorylase genes/pathways have been found in wood-feeding lower and higher termites [6, 13]. However, as shown in Fig. 1a, the two putative GH1 6-phospho-β-glucosidase genes were found clustered together on contig00006 with a phosphoenolpyruvate-dependent phosphotransferase system (PEP-PTS), which in fact is the first reported 6-P-β-glucosidase in termite guts [4, 6, 9, 50]. Compared to two ATP molecules being required for hexokinase generation of glucose-6-phosphate through β-glucosidase hydrolysis, only one ATP is required for each molecule of cellobiose to be metabolized by glycolysis via both cellobiose phosphorylase and 6-P-β-glucosidase pathways (Fig. 3) [70]. Thus, these two latter pathways are recognized as more energy efficient processes for cellobiose metabolism.

Functional validation of these putative enzymes was conducted to test the cellobiose-metabolizing capability of their heterologous proteins (Table 4). Since cloning and characterization of a GH1 β-glucosidase gene (AGS52251) from contig00059 (JQ844187) has been done in our earlier work [37], only 6-P-β-glucosidase and partial putative cellobiose phosphorylase genes were functionally verified here. Heterologous expression of 7 putative GH94 cellobiose phosphorylase genes showed that all members (except *X4_contig00064.27*) could be successfully expressed after IPTG induction, though insoluble inclusion bodies appeared to be the major form (Figure S3). Glucose could be detected by the Glucose (GO) Assay Kit from cellobiose phosphorylase activity assay for the crude enzyme solutions of *X4_contig00091.11*, *X4_contig00064.26*, *X4_contig00057.25*, and *X4_contig00057.26*, while no glucose was detected from that of *X4_contig00023.7* and *X4_contig00068.19*. Heterologous expression of the three putative 6-P-β-glucosidase genes *X4_contig00064.30*, *X4_contig0006.31*, and *X4_contig0006.32* showed that all members could be successfully expressed after induced by IPTG, although insoluble inclusion bodies were the major form (Figure S4). Glucose was detected from 6-P-β-glucosidase activity assay for all the three crude enzyme solutions with Glucose (GO) Assay Kit. Functional verification of these cellobiose metabolic enzymes in the given *Globiterms* termite gut microbiome indicated that diverse cellobiose metabolic pathways were employed by termite gut microbes, to efficiently and rapidly eliminate cellobiose thus minimize its inhibition to upstream cellulases in this tiny intestinal niche.

**Table 4:** Functional verifications of putative cellobiose-metabolizing enzymes

Putative cellobiose-metabolizing enzymes	GH Family	Expression	Activity
β-glucosidase			
X4_contig000059.2	GH1		+^a^
Cellobiose phosphorylase			
X4_contig00023.7	GH94	Supernatent/Percipitation	−
X4_contig00057.25	GH94	Supernatent/Percipitation	+
X4_contig00057.26	GH94	Supernatent/Percipitation	+
X4_contig00064.26	GH94	Supernatent/Percipitation	+
X4_contig00064.27	GH94	No expression	−
X4_contig00068.19	GH94	Supernatent/Percipitation	−
X4_contig00091.11	GH94	Supernatent/Percipitation	+
Phospho-β-glucosidase			
X4_contig00006.31	GH1	Supernatent/Percipitation	+
X4_contig00006.32	GH1	Supernatent/Percipitation	+
X4_contig00064.30	GH4	Supernatent/Percipitation	+

^a^Data from our previous study by Wang et al. [37].

## Conclusion

Termites are only one of a few organisms that are capable of consuming woody biomass as their primary food source [72], and their ecological success stems from their striking ability to rapidly deconstruct their ingested diet. In this study, our integrated genetic and biochemical exploration of the gut metagenome demonstrates a previously unappreciated mechanism of polysaccharide utilization in the unexplored wood-feeding higher termite *G*. *brachycerastes*. This sheds considerable new light on plant cell-wall hydrolysis strategies employed by termite intestinal symbionts. Our results clearly show that highly abundant and diverse fibrolytic gene clusters are present in termite gut bacterial symbionts and these clusters appear to exhibit complementary biochemical features. In particular, the identification of gene clusters containing complete cellulose and hemicellulose cleavage pathways in a single *Treponema* species has not been previously described in termite symbionts. After polysaccharide cleavage, the cellobiose products are predicted to be subjected to a suite of cellobiose-metabolizing pathways to enable flow of glucose toward central metabolism and to relieve the inhibition of hydrolases. In summary, our findings detail important aspects of the termite gut, one of the world’s smallest-yet-efficient bioreactors, which may lead to the development of novel enzyme cocktails for more efficient utilization of plant cell walls.

## Electronic supplementary material

Table S1-S7

Figure S1-S4
