# Supplementary material for: Functional metagenomics reveals abundant polysaccharide-degrading gene clusters and cellobiose utilization pathways within gut microbiota of a wood-feeding higher termite
Source: ISME J. 2018 Aug 16;13(1):104–17. doi: 10.1038/s41396-018-0255-1 (PMC6298952; doi:10.1038/s41396-018-0255-1)

Figure S1. Functional screen of fosmid clones for the four major hydrolases activities including endoglucanase, exoglucanase, β-glucosidase and endoxylanase.

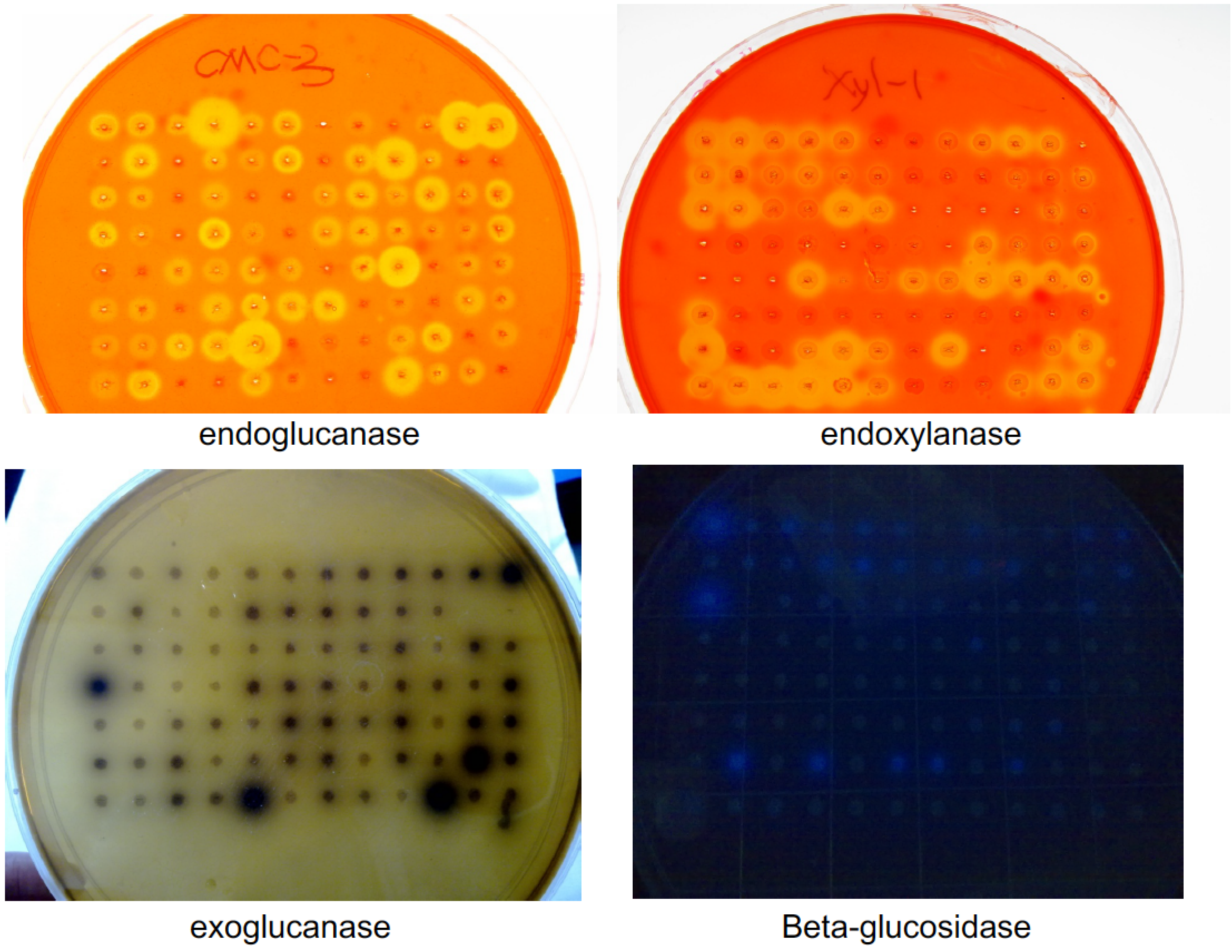

Figure S2. Phylogenetic analysis of main GH families including GH1, GH3, GH5, GH9, GH10, GH11 and GH94 diversity. Multiple sequence alignments were performed with MAFFT against full length of members of GH1, GH3, GH5, GH9, GH10, GH11 and GH94 with each of their top 2 to 6 best hits in the NCBI-nr database. Then an unrooted phylogenetic tree was established in MEGA 5 by the neighbor-joining method with bootstrap values for 1,000 resamplings shown at major nodes. Sequences from the present termite gut metagenome are colored in red, from other termite gut in green and from other various sources in black. Database accession numbers and organisms are showed for each sequence.

Figure S2A. Phylogenetic analysis of GH1 diversity.

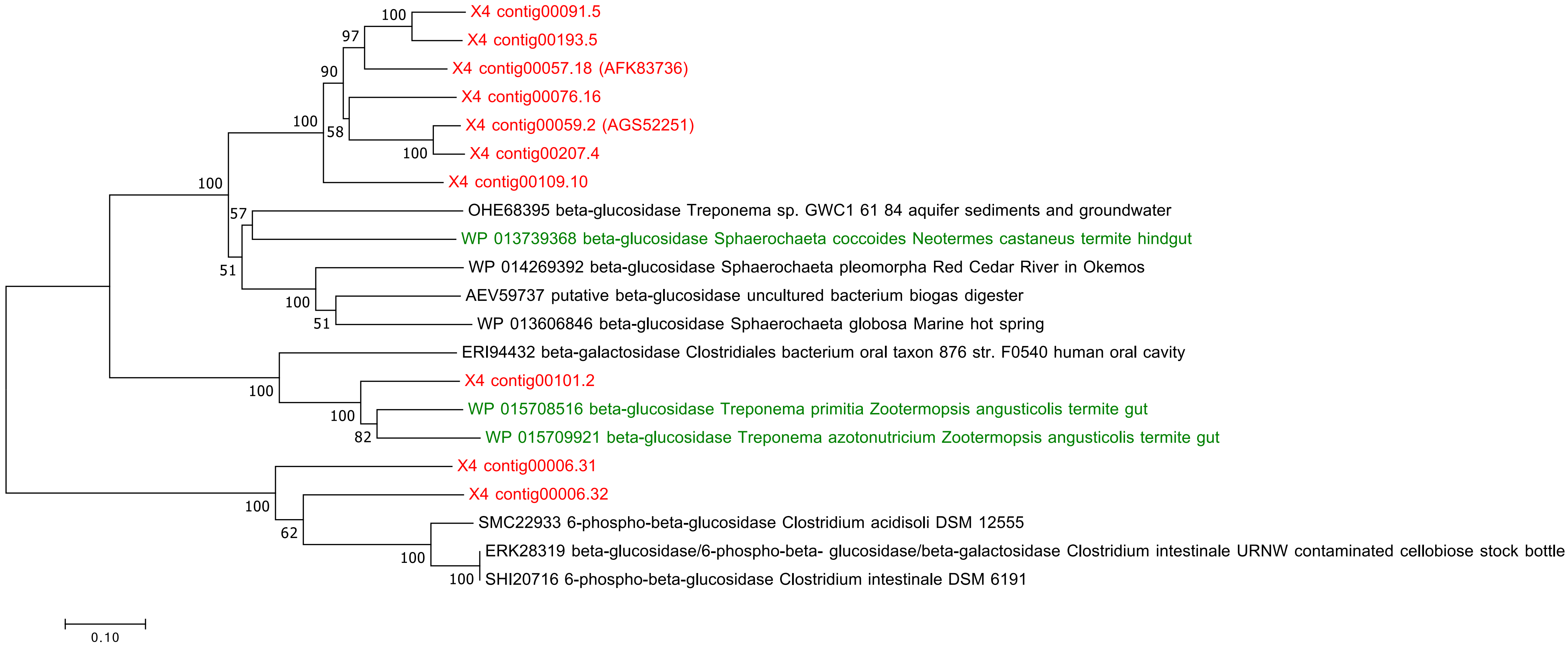

Figure S2B. Phylogenetic analysis of GH3 diversity.

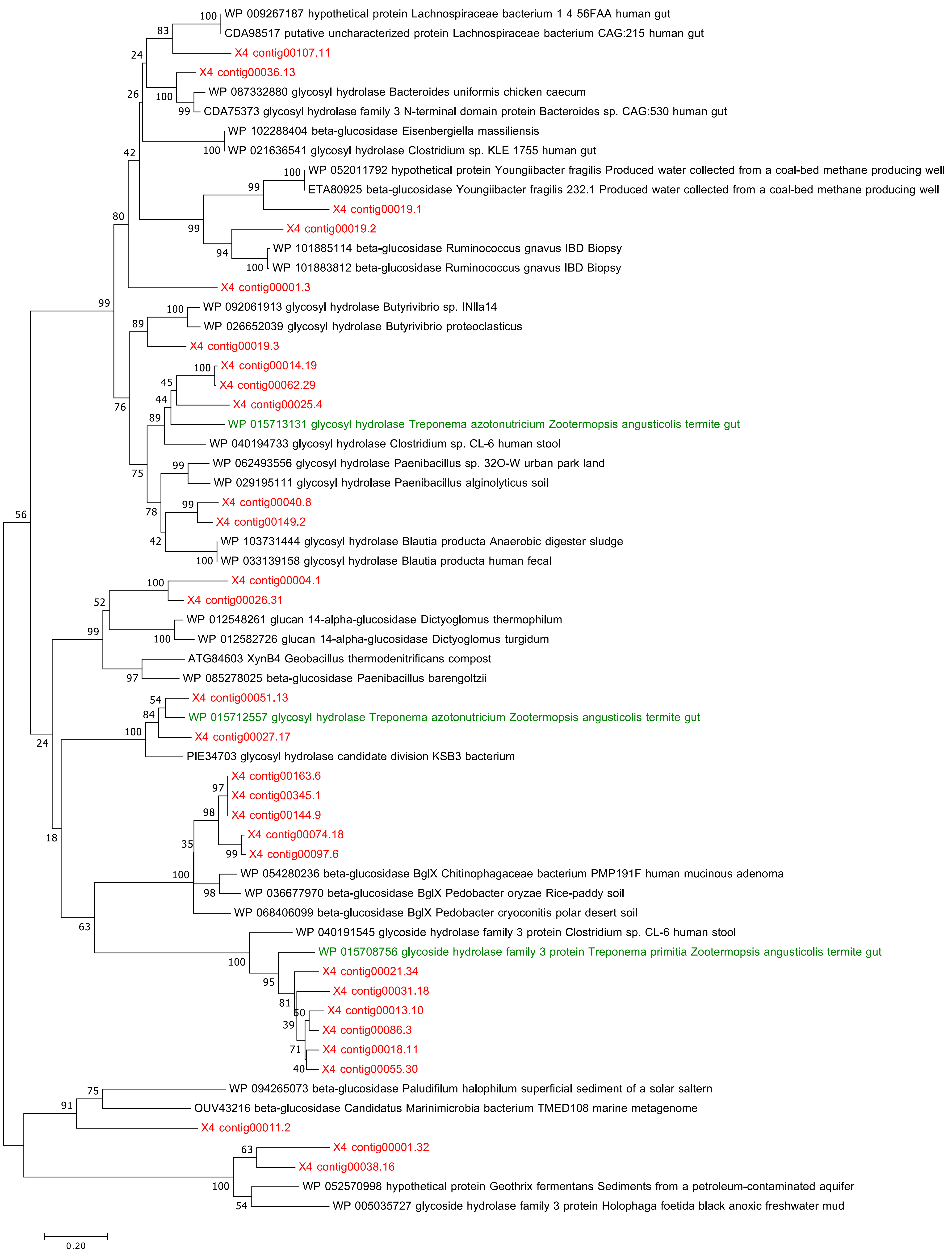

Figure S2B. Phylogenetic analysis of GH3 diversity.

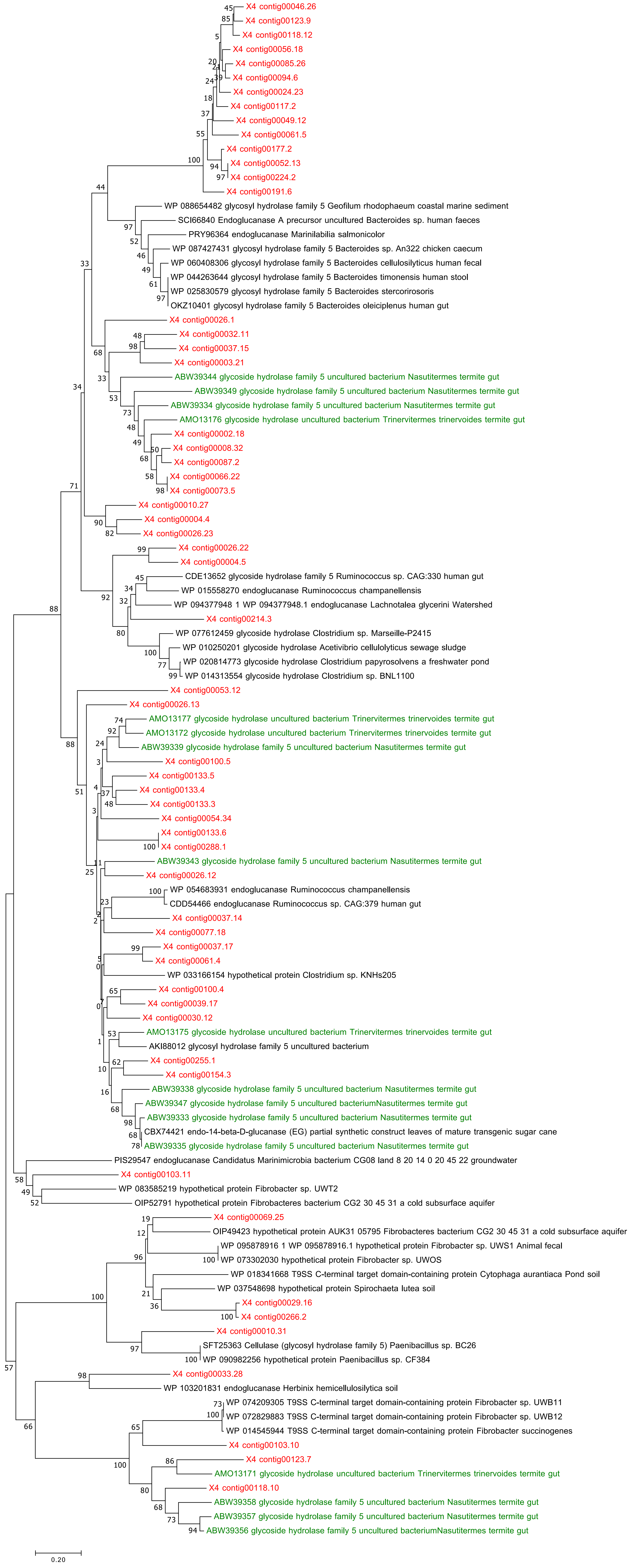

Figure S2D. Phylogenetic analysis of GH9 diversity.

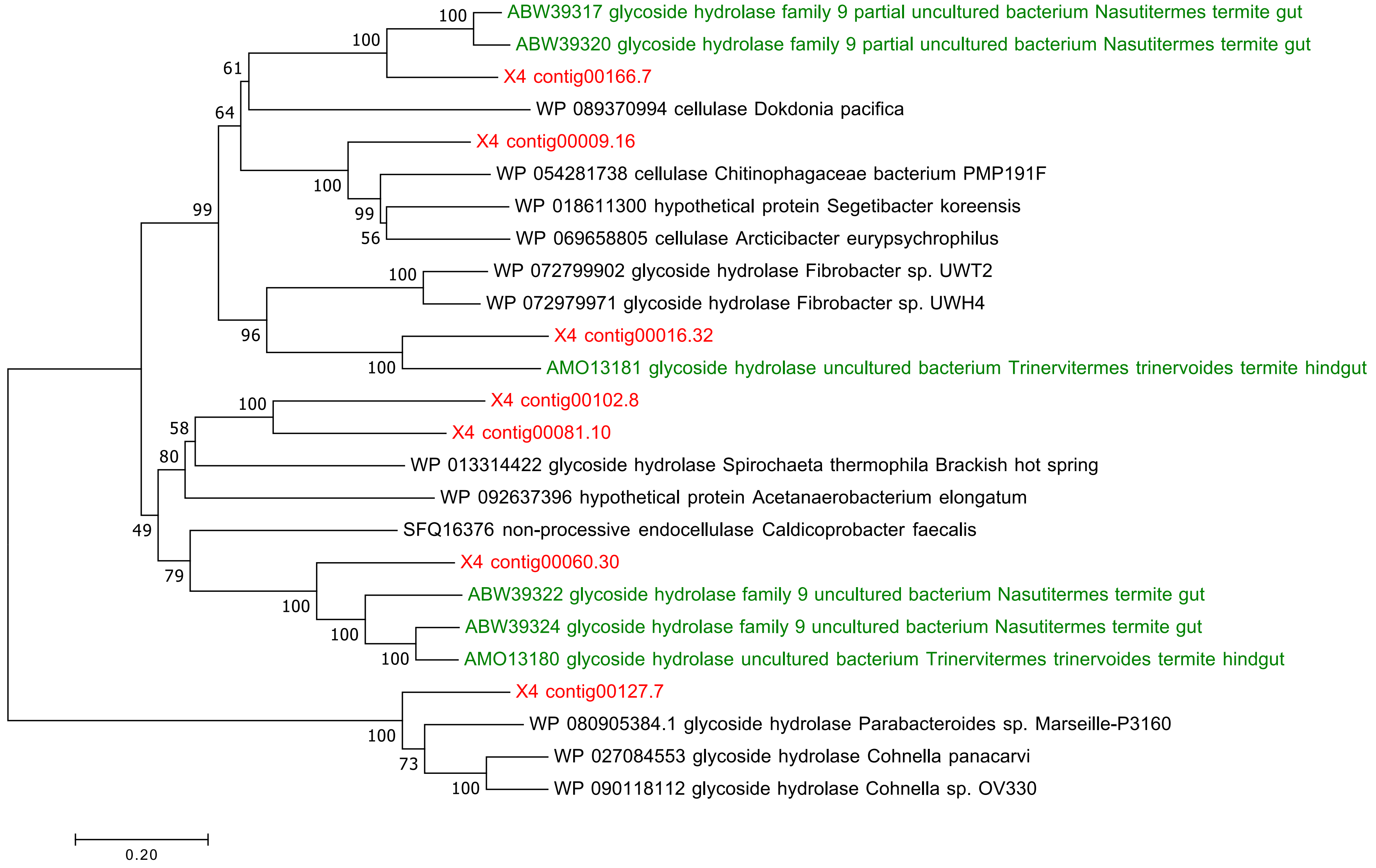

Figure S2E. Phylogenetic analysis of GH10 diversity.

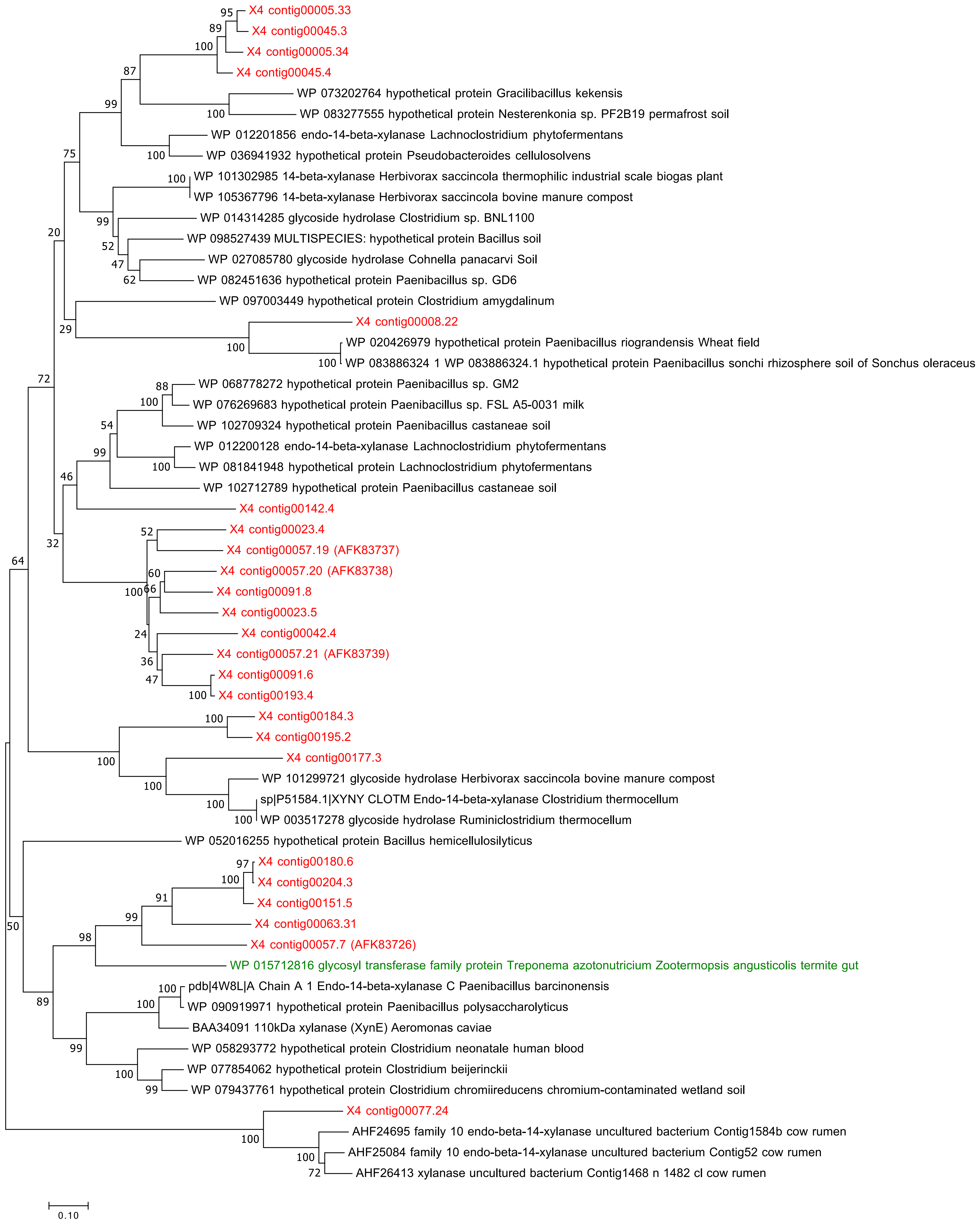

Figure S2F. Phylogenetic analysis of GH11 diversity.

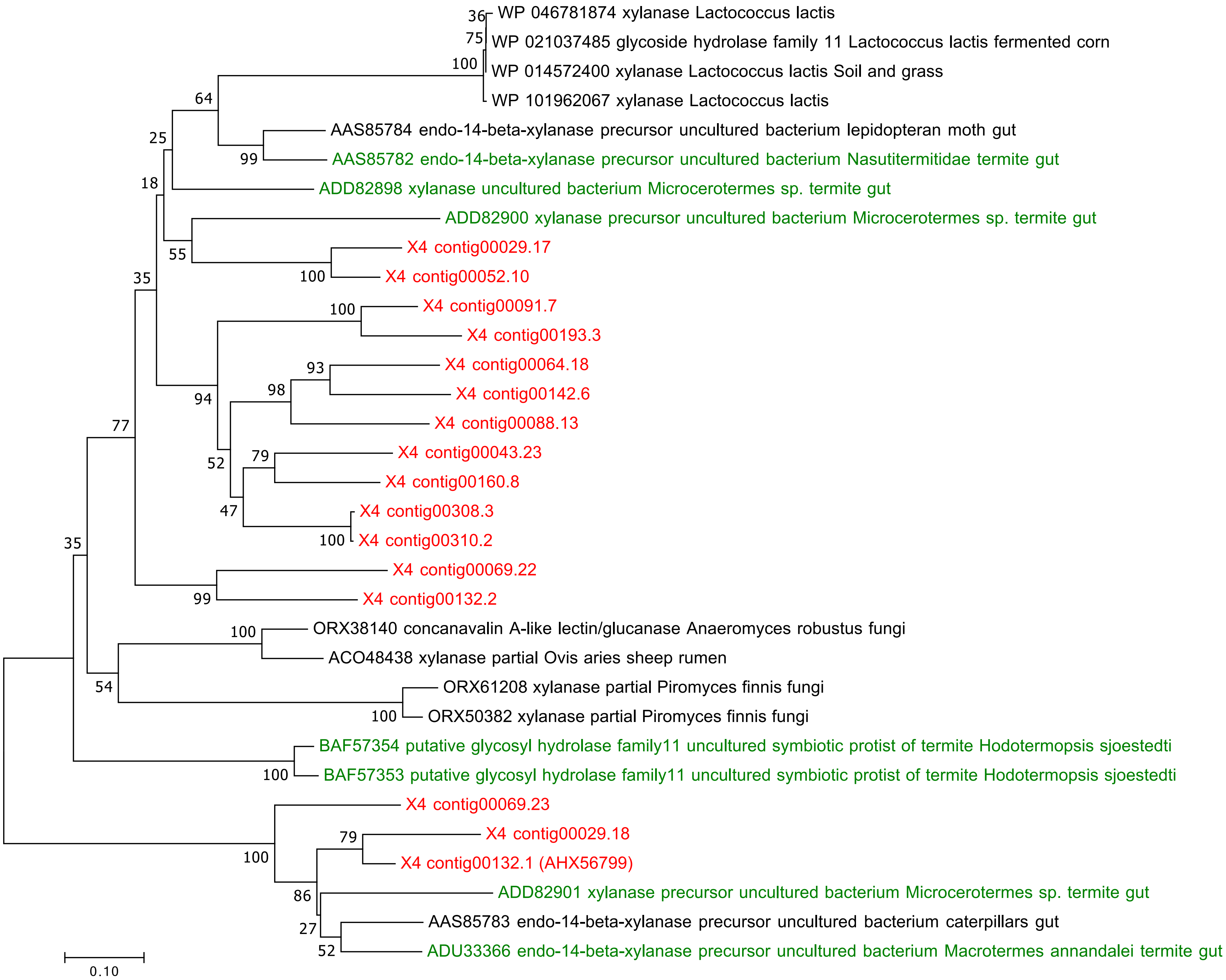

Figure S2G. Phylogenetic analysis of GH94 diversity.

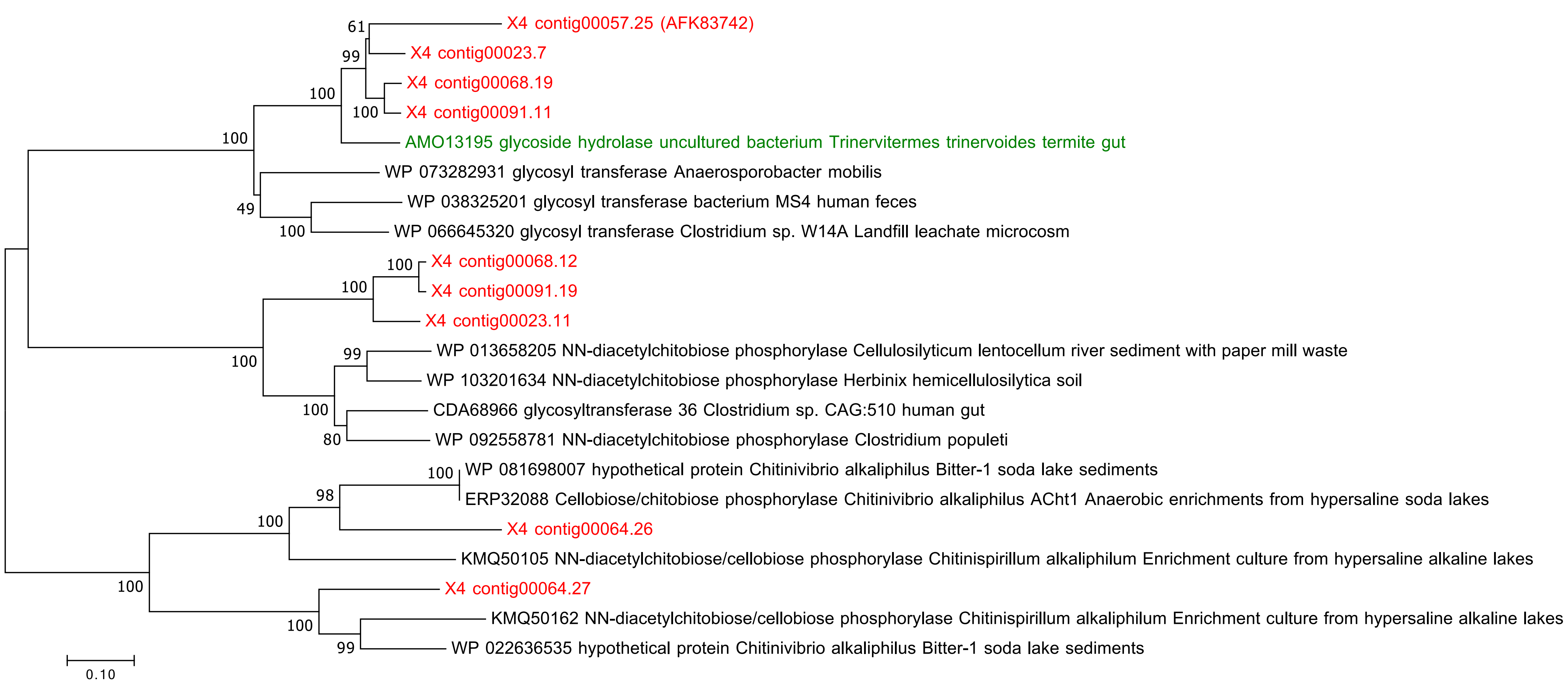

Figure S3. SDS-PAGE analysis of the induction of cellobiose phosphoralase genes. M: stained protein molecular weight marker; Un: total cell protein before induction; In: total cell protein after induction; S: soluble protein fractions in the supernatent of induced cell lysis; P: insoluble protein fractions in the percipitation of induced cell lysis; T: total protein in induced cell lysis. Molecular weight of each enzyme protein is listed below.

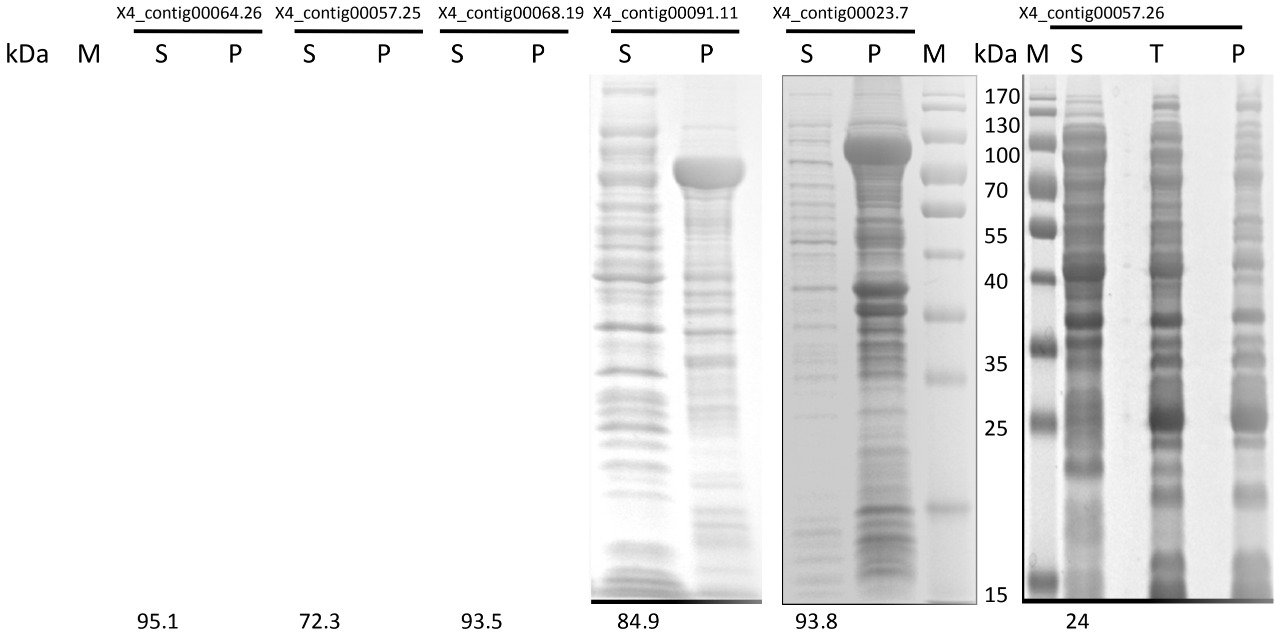

Figure S4. SDS-PAGE analysis of the induction of 6-phospho-β-glucosidase genes. M: stained protein molecular weight marker; T: total protein in induced cell lysis; S: soluble protein fractions in the supernatent of induced cell lysis; P: insoluble protein fractions in the percipitation of induced cell lysis. Molecular weight of each enzyme protein is listed below.

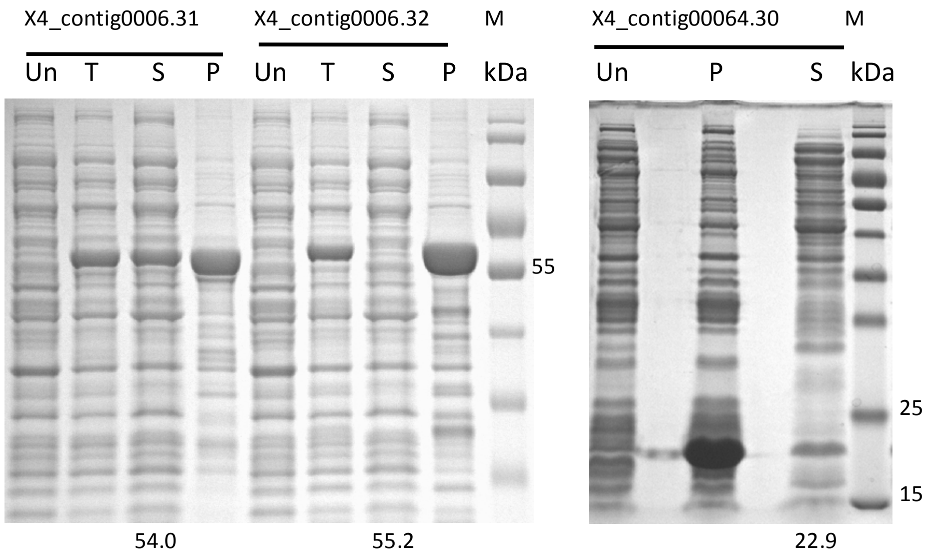

Supplement: Supplementary file 2 — Figure S1-S4 [file 41396_2018_255_MOESM2_ESM.pdf]
